# Three cases of difficulty in achieving definitive loss of consciousness with remimazolam

**DOI:** 10.1186/s40981-021-00485-1

**Published:** 2022-01-11

**Authors:** Mao Miyanishi, Toru Yaguramaki, Yasuhiro Maehara, Osamu Nagata

**Affiliations:** 1 Department of Anesthesiology, The University of Tokyo Hospital, 7-3-1, Hongo, Bunkyo-ku, Tokyo, 113-0033 Japan; 2 Department of Orthopaedic Surgery, JR Tokyo General Hospital, 2-1-3, Yoyogi, Shibuya-ku, Tokyo, 151-8528 Japan; 3 Department of Anesthesiology, Center Hospital of the National Center for Global Health and Medicine, 1-21-1, Toyama, Shinjuku-ku, Tokyo, 162-8655 Japan

**Keywords:** Remimazolam, Benzodiazepine, Tolerance, Bispectral index

## Abstract

**Background:**

Remimazolam is a novel, ultra-short-acting benzodiazepine used for general anesthesia. Because remimazolam is an emerging drug, the tolerance to remimazolam in benzodiazepine-taking patients has been unclear. Also, the efficacy of remimazolam in different races is not fully elucidated so far.

**Case presentation:**

Here we experienced three cases in which high dose of remimazolam was needed for attempting to achieve appropriate anesthetic depth. Two of the three cases were of preoperatively benzodiazepine-taking patients. The other was a case of a Chinese patient. In all three cases, conversion to general anesthesia with propofol was necessitated.

**Conclusions:**

When signs of inadequate sedative effect of remimazolam are observed in patients of benzodiazepine users or of different races, conversion to another sedative agent such as propofol should be considered.

## Background

Remimazolam is a novel, ultra-short-acting benzodiazepine used for general anesthesia, which was launched in Japan in 2020. Remimazolam acts at the benzodiazepine binding site of GABA-A receptors [1]. It has a sedative effect by enhancing the binding of GABA to GABA-A receptors [1].

Remimazolam is rapidly metabolized by carboxylesterase to a metabolite with almost no activity, and an antagonist flumazenil is available [1]. Therefore, rapid awakening is expected. In addition, remimazolam has little circulatory depressant effect [2]. Remimazolam is expected to be used safely in elderly and critically ill patients.

Because remimazolam is an emerging drug, the tolerance to remimazolam in benzodiazepine-taking patients has not been reported in detail. Also, the efficacy of remimazolam in different races is not fully elucidated.

Here we experienced three cases in which high dose of remimazolam was required for adequate anesthesia depth. Two cases were of preoperatively benzodiazepine-taking patients. The other case was of non-Japanese patient. In all cases, conversion to propofol was necessitated.

## Case presentation

### Case #1

A 45-year-old Japanese female (height, 158 cm; weight, 60 kg) was diagnosed with uterine fibroids and scheduled for total hysterectomy. She had been treated for panic disorder with sertraline hydrochloride 50 mg (after breakfast and dinner), escitalopram oxalate 10 mg (after dinner), alprazolam 0.4 mg (after dinner), and bromazepam 3 mg (after dinner). No abnormality was noted in the preoperative examination.

**Fig. 1:**
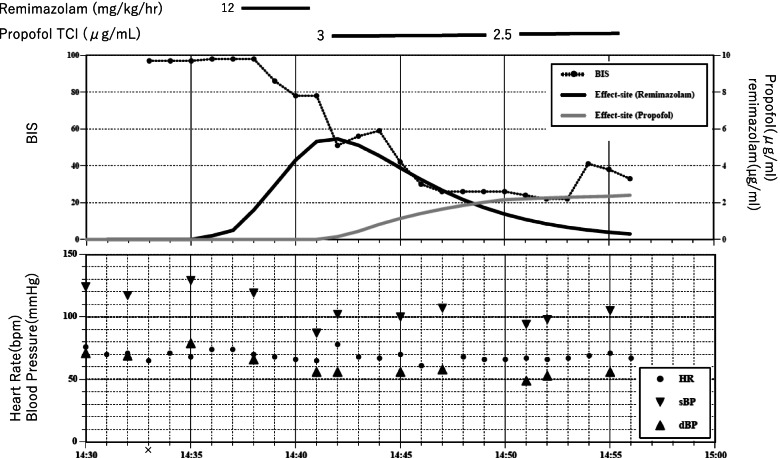
Anesthetic record of case 1. X start of anesthesia. Simulated remimazolam and propofol effect site concentrations are calculated using Doi and Marsh pharmacokinetic models, respectively

On the day of surgery, no oral medication was administered. Oxygen inhalation was started, and remifentanil was administered at 0.5 μg/kg/min. Remimazolam 12 mg/kg/h was started 3 min later (Fig. 1). Within 1 min, eyes did not open in response to a call, and eyelash reflex disappeared. However, over the next 3 min, the upper limbs were in a flexed position, the lower limbs showed spasm-like movements, and BIS remained in the 70s. Even though a total dose of remimazolam 50 mg (0.8 mg/kg) was administered in 4 min, the extremity reactions remained, and BIS did not decrease below 70. Therefore, we gave up anesthesia with remimazolam and switched to propofol. Propofol TCI was started at 3.0 μg/ml (propofol 42 mg was administered as an initial dose). One minute later, BIS decreased to the 30s, and limb movements disappeared. Rocuronium 50 mg was administered and tracheal intubation was performed. Intraoperative anesthesia was maintained with propofol 2.5 μg/ml and remifentanil 0.5 μg /kg/min. After surgery, propofol and remifentanil were terminated. She responded to calling after 8 min, and she was extubated. She had no memory of the anesthesia induction.

### Case #2

A 46-year-old Japanese female (height, 166 cm; weight, 63 kg) was diagnosed with uterine fibroids and scheduled for total hysterectomy. From 15 years ago, she has been treated for post-traumatic stress disorder with escitalopram oxalate 10 mg, ethyl loflazepate 1 mg, domperidone 5 mg, and suvorexant 20 mg before bedtime. No abnormality was noted in the preoperative examination.

**Fig. 2:**
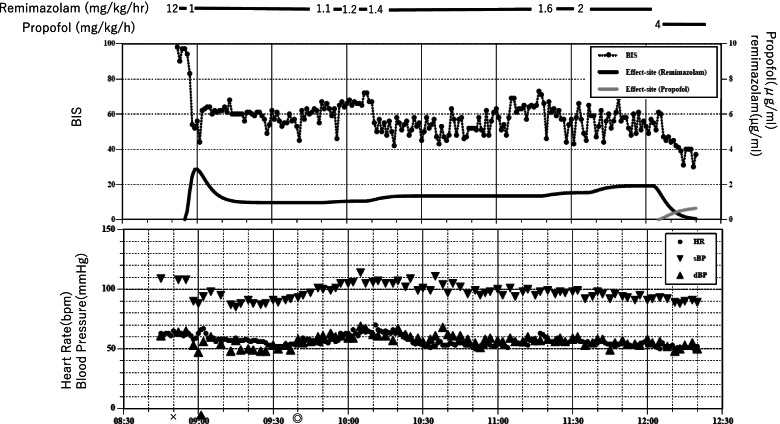
Anesthetic record of case 2. X start of anesthesia, ▲ tracheal intubation, ◎ start of surgery. Simulated remimazolam and propofol effect site concentrations are calculated using Doi and Marsh pharmacokinetic models, respectively

After entering the operating room, oxygen inhalation was started, and remifentanil 0.5 μg/kg/min was administered. Remimazolam 12 mg/kg/h was started 3 min later, and her response to call and eyelash reflex disappeared in 90 s (Fig. 2). BIS at this time was 56. Remimazolam was reduced to 1 mg/kg/h. Tracheal intubation was performed after administration of rocuronium 50 mg. Immediately after reducing the dose of remimazolam to 1mg/kg/h, BIS was in the 60s, and after 53 min, it reached the 70s. Therefore, remimazolam was increased to 1.1 mg/kg/h. BIS temporarily remained in the 60s, but increased to the 70s after 14 min. Therefore, the dose of remimazolam was increased to 1.4 mg/kg/h. BIS remained in the 40s to 60s for a while, but gradually increased to 76 in 71 min, and the remimazolam was increased to 1.6 mg/kg/h. Once BIS settled at the 50s, it gradually increased and rose to 66 after 19 min. Thus, the remimazolam was increased to 2 mg/kg/h. However, no BIS decrease was observed. After 25 min, continuous administration of remimazolam was abandoned and changed to anesthesia with propofol. Subsequent anesthesia was maintained with propofol 3–4 mg/kg/h and remifentanil 0.2–0.6 μg/kg/min. BIS remained stable in the 40s. After surgery, propofol and remifentanil were terminated, and sugammadex was administered at 2 mg/kg. She responded to calling 12 min later, and she was extubated. During the administration of remimazolam, there were no changes in pulse or blood pressure and no signs of arousal. She did not remember anything during the anesthesia.

### Case #3

A 44-year-old Chinese female (height, 162 cm; weight, 54 kg) suffered a fracture of the right tibial plateau in a traffic accident and was scheduled for hematopoietic surgery. There were no previous medical conditions or medications. There was no abnormality in the preoperative examination.

**Fig. 3:**
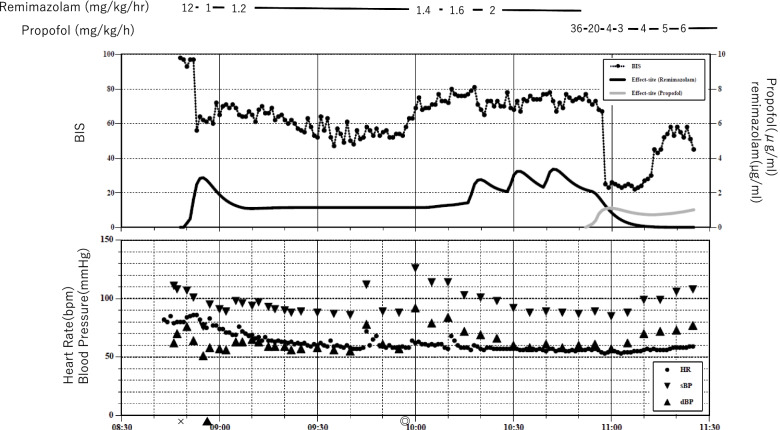
Anesthetic record of case 3. X start of anesthesia, ▲ tracheal intubation, ◎ start of surgery. Simulated remimazolam and propofol effect site concentrations are calculated using Doi and Marsh pharmacokinetic models, respectively

After entering the operating room, oxygen inhalation was started, and remifentanil 0.5 μg/kg/min was administered. Remimazolam 12 mg/kg/h was started 2 min later (Fig. 3). The response to call and eyelash reflex disappeared in 2 min. BIS at this time was 56. Remimazolam was reduced to 1 mg/kg/h, and tracheal intubation was performed after administration of rocuronium 40 mg. Then, a sciatic nerve block was performed at distal thigh level. As BIS remained in the 60s immediately after the reduction of remimazolam, remimazolam was increased to 1.2 mg/kg/h after 16 min. BIS temporarily dropped to around 50, but rose to the 60s again, and thus, remimazolam was increased to 1.4 mg/kg/h. BIS did not decrease and rose to the 70s temporarily, so remimazolam was increased to 1.6 mg/kg/h 8 min later. However, no BIS decrease was observed after that. An administration of a bolus of remimazolam 0.2 mg/kg (10 mg) showed no change in BIS, so remimazolam was increased to 2 mg/kg/h after 11 min. After that, even after two additional boluses of remimazolam 0.2 mg/kg, BIS did not decrease. After 32 min, we abandoned the continuous administration of remimazolam and changed to propofol. After a bolus of propofol 30 mg was administered as an initial dose, BIS promptly decreased to the 20s. Propofol 6 mg/kg/h and remifentanil 0.25–0.5 μg/kg/min were used to maintain anesthesia, and BIS remained stable in the 30–50 range. After the surgery, propofol and remifentanil were terminated. The patient was extubated 15 min after the termination of propofol and remifentanil. During the administration of remimazolam, there were no changes in pulse or blood pressure and no signs of arousal. She did not remember anything during anesthesia.

## Discussion

In cases 1 and 2, remimazolam was administered in patients using benzodiazepine preoperatively. In case 1, although the patient stopped responding to calls after starting remimazolam administration, BIS remained high and extremity movements continued for 4 min. In case 2, BIS exceeded 60 even at the maximum dose rate of 2 mg/kg/h of remimazolam. Several studies have identified neuroadaptive mechanisms underlying benzodiazepine tolerance including alterations in GABA-A receptor subunit mRNA expression [3, 4]. However, the details of these mechanisms have not been fully elucidated. In these cases, the tolerance to benzodiazepines may be contributed to the inadequate sedative effect of remimazolam.

Case 3 was a case of general anesthesia with remimazolam in an ethnic group different from Japanese. BIS exceeded 60 even when using remimazolam 2 mg/kg/h. In general, racial factors that affect drug efficacy include genetic polymorphisms of drug-metabolizing enzymes, receptor sensitivity, pharmacokinetics, or medical practices. In regard to remimazolam, the effects of racial differences on these factors have not been known. In a comparison of remimazolam and propofol as sedatives during colonoscopy in Chinese patients, it was reported that the sedative effect of remimazolam was non-inferior [5]. Further investigation is warranted.

We used BIS to estimate the depth of anesthesia with remimazolam. The algorithm for calculating BIS is mainly adjusted for propofol, and the reliability of BIS for benzodiazepines has been questionable. BIS in patients sedated with midazolam was reported to be significantly different between responders to voice and nonresponders [6]. As for remimazolam, in a Phase I study, remimazolam caused 95% and 50% of subjects to lose consciousness, with BIS values of 54 and 66, respectively, suggesting that BIS can be used to assess the state of consciousness [7]. However, reliability of BIS for remimazolam has not been reported in detail. Therefore, in order to estimate anesthesia depth with remimazolam, it is important to consider not only BIS but also body movements, vital signs changes, regular medications, or race.

In these cases, BIS decreased rapidly after changing from remimazolam to propofol. Propofol and benzodiazepines bind to different subunits of GABA A receptor. Furthermore, propofol acts on many sites besides GABA A receptors, such as NMDA receptors [8]. Therefore, adequate sedation may be provided by propofol in patients who were insufficiently sedated with remimazolam.

We experienced three cases of inadequate sedation with remimazolam. When high BIS values or limb movements are observed during anesthesia with remimazolam in patients taking benzodiazepine preoperatively or of different races, it is considered safer to switch to other sedatives.

## Data Availability

Not applicable

## Abbreviations

GABA: Gamma-aminobutyric acid
BIS: Bispectral index
TCI: Target-controlled infusion
TOFC: Train of four count
HR: Heart rate
sBP: Systolic blood pressure
dBP: Diastolic blood pressure
